# Suppurative Pericarditis

**Published:** 1901-01-05

**Authors:** 


### SUPPURATIVE PERICARDITIS.

AMONG the papers read at the last meeting of the Clinical Society was one by Dr. John Fawcett and Mr. F. J. Shaw on a case of pneumococcal suppurative pericarditis treated by incision and drainage. The patient was a boy aged eight years, who was admitted to Guy's Hospital six days after the commencement of his illness. There were then signs of lobar pneumonia at the right base. The next day signs of pericarditis were noted, and ten days later the pericardium was explored, as from the extent of the effusion and the boy's general condition it was thought that the effusion was possibly purulent. Among other signs there was marked tenderness of the epigastrium on pressure. Pus having been found on exploration, the pericardium was incised and drained by the removal of a portion of the fifth left costal cartilage. After the operation the boy's condition much improved, but a fortnight later a small empyema of streptococcal origin was found on the right side, and he died. At the necropsy it was found that the condition of the pericardium was rapidly clearing up, and it seemed almost certain that had not the boy developed a streptococcal empyema he would have survived. It is worth noting that no friction sound was heard in this case, although it had been frequently listened for. In the discussion on this paper the question of the sort of anæsthetic to be used in such cases arose, Mr. F. C. Wallis saying that he thought that local anæsthesia was most advisable. General anæsthesia had, he said, been very fatal in such cases. He regarded eucaine as decidedly preferable to cocaine, for the latter had a distinctly toxic effect which was not observed with eucaine. He had used as much as four drachms of a 4 per cent. solution of eucaine without evil effects, and had even excised rib under its influence. Mr. Arthur Barker agreed as to the value of eucaine and local anæsthesia, but thought that the emotional condition of children must not be forgotten, for notwithstanding the absence of pain a certain fortitude was required on the part of the patient. To this Mr. Wallis replied that he had adopted the method of blindfolding the children and using local freezing for the needle puncture.

